# Towards the Development of a Low Cost Airborne Sensing System to Monitor Dust Particles after Blasting at Open-Pit Mine Sites

**DOI:** 10.3390/s150819667

**Published:** 2015-08-12

**Authors:** Miguel Alvarado, Felipe Gonzalez, Andrew Fletcher, Ashray Doshi

**Affiliations:** 1 Centre for Mined Land Rehabilitation, Sustainable Mineral Institute, The University of Queensland, Brisbane 4072, Australia; 2 Science and Engineering Faculty, Queensland University of Technology (QUT), Brisbane 4000, Australia; E-Mail: felipe.gonzalez@qut.edu.au; 3 Centre for Mined Land Rehabilitation, Sustainable Mineral Institute, The University of Queensland, Brisbane 4072, Australia; E-Mail: a.fletcher@cmlr.uq.edu.au; 4 Faculty of Engineering, Architecture and Information Technology, School of Information Technology and Electrical Engineering, The University of Queensland, St. Lucia 4072, Australia; E-Mail: ashraydoshi@gmail.com

**Keywords:** PM10, monitoring, blasting, fixed-wing UAV, quadcopter, optical sensor

## Abstract

Blasting is an integral part of large-scale open cut mining that often occurs in close proximity to population centers and often results in the emission of particulate material and gases potentially hazardous to health. Current air quality monitoring methods rely on limited numbers of fixed sampling locations to validate a complex fluid environment and collect sufficient data to confirm model effectiveness. This paper describes the development of a methodology to address the need of a more precise approach that is capable of characterizing blasting plumes in near-real time. The integration of the system required the modification and integration of an opto-electrical dust sensor, SHARP GP2Y10, into a small fixed-wing and multi-rotor copter, resulting in the collection of data streamed during flight. The paper also describes the calibration of the optical sensor with an industry grade dust-monitoring device, Dusttrak 8520, demonstrating a high correlation between them, with correlation coefficients (R^2^) greater than 0.9. The laboratory and field tests demonstrate the feasibility of coupling the sensor with the UAVs. However, further work must be done in the areas of sensor selection and calibration as well as flight planning.

## 1. Introduction

The mining and coal seam gas industries in Australia and around the world are important economic activities. Coal exports from Queensland from March 2013 to March 2014 totaled more than $24.5b [1]. These activities generate particles and gases such as methane (CH_4_), carbon dioxide (CO_2_), nitrogen oxides (NO_x_), and sulfur oxides (SO_x_) that have potentially dangerous environmental and health impacts.

Blasting in particular includes effects such as airblast, ground vibration, flyrock, toxic gases and particulate matter [2,3]. Particulate matter, aerosols, ammonia, carbon dioxide (CO_2_), nitrogen, nitrogen oxides (NO_x_) and sulfur oxides (SO_x_) are the primary residues produced by blasting events at mining sites. In an ideal situation, the exothermic reaction produces CO_2_, water vapor and molecular nitrogen (N_2_); however, due to environmental and technical factors, other noxious gases are often produced in a range of concentrations [4].

In this paper, we propose the use of small unmanned aerial vehicles (UAV) carrying air quality sensors to allow precise characterization of blasting plumes in near-real time. This approach may lead to actionable data for harm avoidance or minimization. Most pollution dispersion models use predefined estimates of pollution sources and atmospheric conditions; near-real time information from within the plume has been practically impossible to collect. Flight instrument data transmitted as telemetry from the UAV provides high resolution instantaneous micrometeorological data that can assist interpretation of concentrations detected by on-board air quality sensors. In addition, this information including location, micrometeorological data and air quality, can be delivered in real time to analytical software. The data stream may therefore be used to feed flight path-planning algorithms or atmospheric dispersion models in near-real time.

In order to assess this approach, fixed-wing and multi-rotor UAVs were used. These UAVs were developed at The University of Queensland for ecological investigations. The platforms were capable of autonomous predetermined flight path planning or semi-autonomous direction. These platforms have weight restrictions and require sensors with high temporal sampling resolution (<1 s that can be digitally sampled but allow air quality sensors to be integrated and tested. In this paper we tested light-emitting diode (LED)-based optical sensors due to the combination of essential characteristics including rapid response, light weight and ease of data digitization. To date, two dust sensors have been tested with the UAV (SHARP GP2Y10 and Samyoung DSM501A) [5,6].

Characterizing blasting plumes and predicting dispersion using this approach requires integration of a number of factors:

- Development or modification of micro UAV platforms that can be safely operated near active mine blasts.
- Identification of sensors with necessary sampling rates (<1 s), weight (<500 g), data output format and sufficient sensitivity (1 mg/m^3^ PM10).
- System endurance sufficient to capture plume evolution and dynamics (>20 min).
- Integration and formatting of data streams necessary for mathematical predictive models via live telemetry.

This project aims to develop tools that inform, cross calibrate and validate plume models for particulate and gaseous pollutants associated with blasting activities.

This paper is organized as follows: Section 2 reviews current methods to monitor blasting plumes, dust and gases after blasting at open-cast mine sites, and the use of UAVs for environmental monitoring and modeling approaches; Section 3 describes the current sensing system that has been developed; Section 4 describes progress in the integration of the dust sensor system with UAVs and flight testing; and, Section 5 outlines current conclusions and further work.

## 2. Blast-Associated Air Sampling

### 2.1. Methods to Monitor Blasting Plumes

Blast-associated dust is a significant potential hazard, and novel monitoring methods are continuously explored. Roy *et al.* developed a multi-platform system using ground-based dust samplers in combination with balloon-carried samplers near open pit mines. The data collected informed multiple regression and neural network models how to monitor and predict the drifting of blast plumes [7,8]. As samplers were static during blasting, this approach required detailed site-blasting plans and favorable weather conditions to determine their interconnectivity. Under this configuration, neural network models performed better than multiple regression models in predicting outcomes [9].

Furthermore, fugitive NO_2_ and PM10 emissions of coal mining in the Hunter Valley, Australia have been examined using gravimetric and LIDAR methods. LIDAR provided long-path laser-integrated concentration signal with very low limit of detection, but required a fixed location [10]. Attalla *et al.* used a different approach by implementing NDIR (non-dispersion infrared) and mini-DOAS (differential optical absorption spectroscopy) for prediction of NOx and other pollutant gases. This method also required a fixed-location ground-based sensing apparatus. Modelling in AFTOX (Air Force toxics model) resulted in overestimation of plume concentrations at a distance [4].

Richardson (2013) assessed particulate fractions using a scintillation probe dust sensor (Environmental Beta Attenuation Monitors—EBAMs) and a real-time laser photometer (Dusttrak) in Hunter Valley and Central Queensland (Goonyella Riverside) and confirmed that PM2.5 is a small fraction of the overall suspended blast-associated particles, while PM10 is dominant [11].

### 2.2. Dust Sampling Sensors

The method and type of sensors used to measure contaminant gas or dust emissions will vary according to the type of emission, concentration range of concern, and required response time. Sensors are commonly based on ultrasound, optical, and electrochemical sensing elements [12,13,14]. These sensors can either be handheld, installed in vehicles, or form ground-based network systems. Table 1 shows different examples of sensors and their characteristics classified by the way they are implemented. Network systems are very useful when specific receptors or areas are to be monitored [15,16]. However, effective monitoring diameter, costs of installation, operation and maintenance are important considerations that may limit their use and procurement.

**Table 1:** Example of sensing technology used for monitoring gases in the mining, oil and gas industries.

Instrument	Description	Gases/Particles	Characteristics
***Handheld***			
Dräger X-am 5600 [17]	Compact instrument for the measurement of up to 6 gases; complies with standard IP67; IR sensor for CO_2_ and electrochemical for other gases.	O_2_, Cl_2_, CO, CO_2_, H_2_, H_2_S, HCN, NH_3_, NO, NO_2_, PH_3_, SO_2_, O_3_, Amine, Odorant, COCl_2_ and organic vapors.	Dimensions: 4.7 × 13.0 × 4.4 cm Weight: 250 g
***Installed in ground vehicles***			
Picarro Surveyor [18,19]	Cavity ring-down spectroscopy (CRDS) technology, sensitivity down to parts-per-billion (ppb); survey gas at traffic speeds and map results in real time; real-time analysis to distinguish natural gas and other biogenic sources.	CO_2_, CO, CH_4_, and water vapor	Dimensions: Analyzer 43.2 × 17.8 × 44.6 cm; external pump 19 × 10.2 × 28.0 cm Weight: 24 kg + vehicle Power: 100–240 VAC
***Stationary***			
Tapered Element Oscillating Microbalance (TEOM) [20,21].	Continuous particle monitoring. The tapered element consists of a filter cartridge installed on the tip of a hollow glass tube. Additional weight from particles that collect on the filter changes the frequency at which the tube oscillates.	Total suspended particles (TSP), PM10, PM2.5	Dimensions: 43.2 × 48.3 × 127.0 cm) Weight: 34 kg Power: 100–240 VAC
***Networks***			
AQMesh [22]	Wireless monitor; high sensitivity (levels to ppb); designed to work through a network of arrayed monitors.	NO, NO_2_, O_3_, CO, SO_2_, humidity and atmospheric pressure.	Dimensions: 17.0 × 18.0 × 14.0 cm Weight: <2 kg Power: LiPo batteries
***Airborne***			
Yellow scan [23]	LIDAR technology with a total weight of 2.2 kg; 80,000 shots/s; resolution of 4 cm; class 1 laser at 905 nm.	Dust and aerosols.	Dimensions: 17.2 × 20.6 × 4.7 cm Weight: 2.2 kg Power: 20 W

A complex criteria matrix must be considered when selecting an airborne sensor to monitor blast plumes. Factors include dimensions (weight and size); tolerance of vibration and movement given mounting on a UAV platform (up to 15 m/s); concentration range of sensor as well as the accuracy and limitations of the sensor (e.g., response time, mean square deviation, calibration, interference of other gases, humidity and temperature).

Optical LED particulate sensors are potentially suitable devices that could be used to explore the proposed system. LED sensors have the advantages of low power consumption, high durability, compact size, and easy handling and have been tested as a reliable source light for DOAS [24]. They also have demonstrated the ability to reduce internal stray light and can be used as a light source for applications requiring numerous kilometers of total light path [25]. Several other authors have also highlighted their advantages over other types of sensors [26,27].

### 2.3. Use of Unmanned Aerial Vehicles (UAVs) for Environmental Monitoring

Researchers are identifying advantages of UAVs to undertake investigations in difficult terrain/landscape areas, where health and safety risks exist, or where there is a lack of resources (human and/or economic). Gas sensing with small-micro UAVs using electrochemical and optical sensors is still not well established due to rapid development of sensors and UAVs. Sensing of CO_2_, CH_4_ and water vapor [28], NO_2_ and NH_3_ [29], ethanol and CH_4_ [30,31], have been conducted using rotary-winged platforms. Watai *et al.* [32], used a kite plane to monitor CO_2_ using a NDIR gas analyzer which had a response time of 20 s. A spectrum-specific video camera has been developed to visualize SO_2_ emissions from volcanoes by Brown *et al.* [33]. Lega *et al.* integrated a multi-rotor-sensing platform that monitors air pollutants in real time and provides 3D visualization [34]. Several variations of this platform, StillFly and BiLIFT, detect gases like CO, C_6_H_6_, NO_2_, O_3_, SO_2_, NO_X_ and PM10, as well as thermal IR images to detect sewage discharges along the coastline of Italy [34,35]. Fixed-wing systems capable of achieving real time monitoring and providing indexed-linked samples are also currently possible [34]. Target sampling locations and source scales will be important to platform and sensor selection due to the fundamental differences between fixed-wing and multi-rotor UAVs, such as hovering capacity, endurance, and flight envelope.

Other approaches have been taken to characterize and track fugitive emission contamination plumes and register their concentrations [4,7,29,36,37,38,39]. However, to the authors’ knowledge, UAVs have never been used to understand dust or gas emission associated with mine blasting. Small UAV platforms and real-time air quality sampling impose a number of novel and complex sampling requirements that still need to be addressed:

- Rapid sensor response time is important in mobile sensing platforms that move relative to both air and ground.
- Limited power requires flight efficiency and sensors with low power consumption.
- Sensors that require extended duration equilibration times (~20 s) require sampling chambers, and delayed response times result in difficult or impossible flight performance required to return to the estimated plume location.
- Multi-rotor platforms operate via GPS, and thus approximate a ground-based sampler independent of the fluid it is sampling. Fixed-wing platforms move through a defined volume of air in a given time regardless of ground location.
- Moving platforms require flow control for sample chambers to ensure calibrated values can be reported.
- The use of mathematical models is an essential element when monitoring air quality and atmospheric contamination. Defining appropriate models given the type of data collected is important. There are several approaches commonly used for air (emission factors, Gaussian, Lagrangian, Eulerian, *etc.*), each of which have limitations to their performance [40,41,42].
- Improve data visualization since current attempts to map pollutants in the atmosphere are presented as snapshots, not as a dynamic environment with concentration measurements that change before and after the moment a reading is produced.

## 3. Design of Sensing System

The sensor system consists of a gas-sensing node, the UAV, and a data integration and visualization interface.

### 3.1. System Architecture

**Figure 1:**
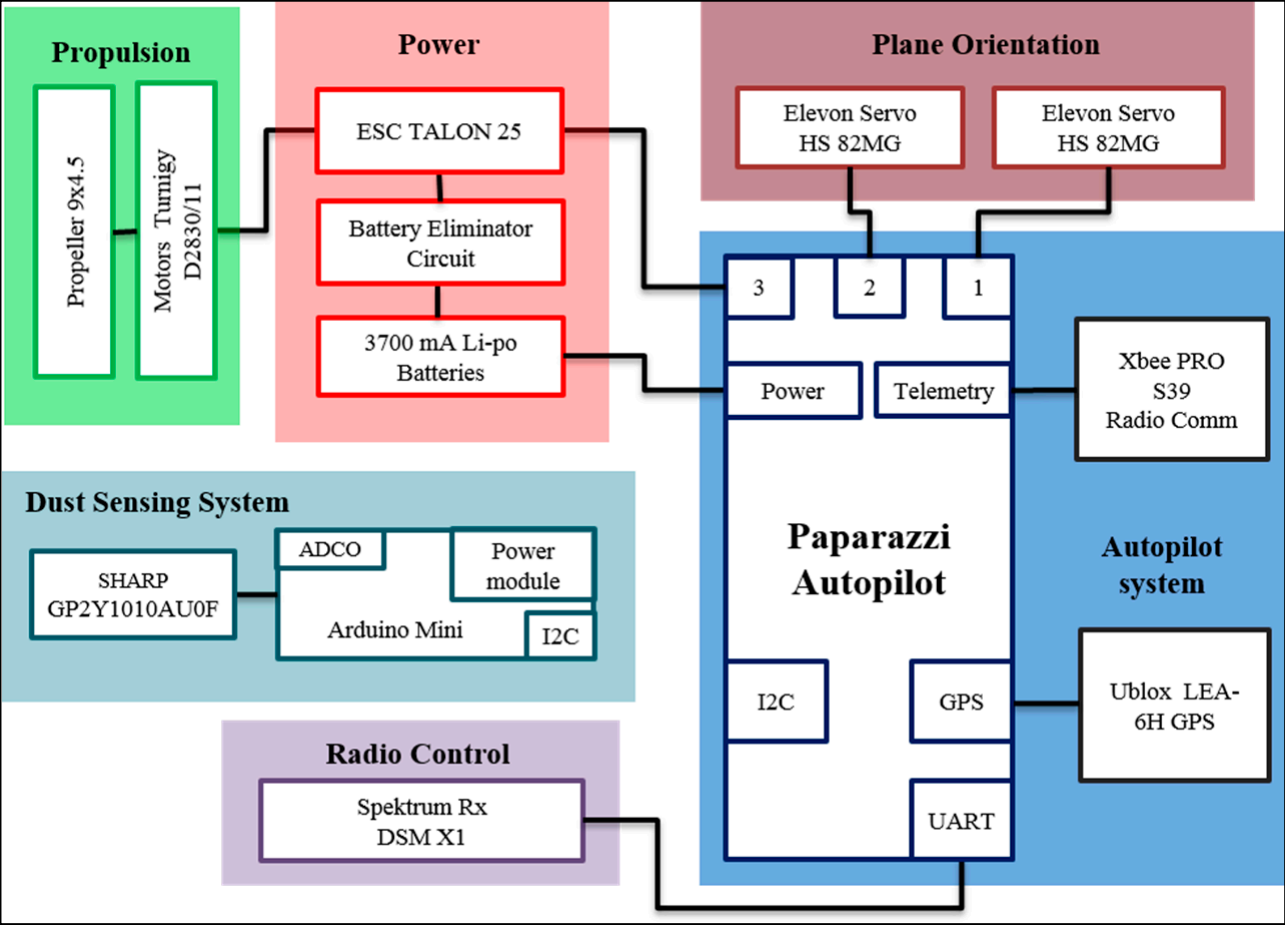
System architecture for the fixed-wing UAV with dust sensor.

**Figure 2:**
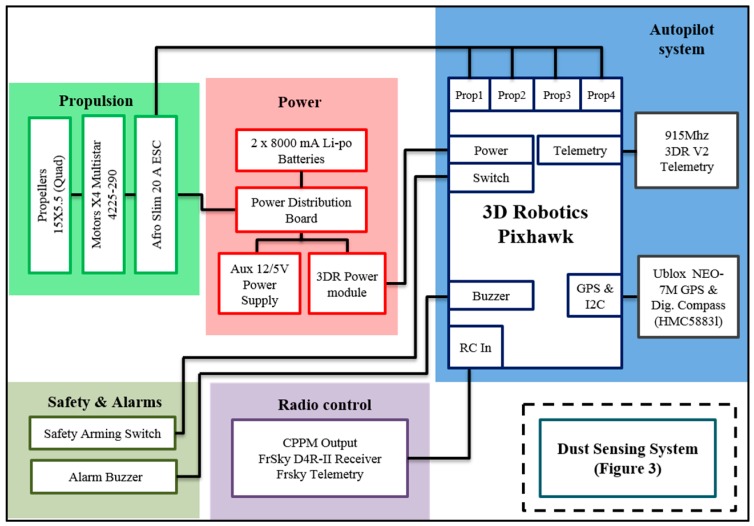
System architecture for quadcopter UAV with independent gas-sensing system.

The system architecture for the fixed-wing UAV with integrated dust sensor (Figure 1) and a multi-rotor carrying a telemetered dust sensor (Figure 2) are necessarily different due to the use of different autopilots. Micro meteorological data deduced from UAV platform flight control is a novel and detailed source of data for interpretation of air quality measurements. However, it requires integration with gas sensor data to allow a meaningful application.

### 3.2. Gas-Sensing Node

**Figure 3:**
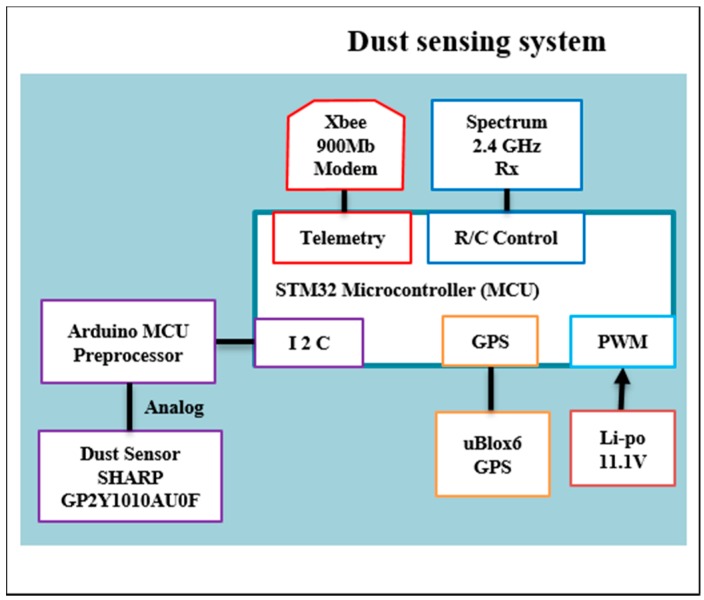
System architecture for the modular dust sensor.

Two sensors were experimentally assessed to date: GP2Y10 (SHARP) and DSM501A (Samyoung) for PM10. The SHARP and Samyoung sensors tested were connected through an Arduino microcontroller to integrate sensor-telemetry data streams. The sensor and associated electronics are low weight and constrained size to allow simple installation in other multi-rotor or fixed-wing platforms. The dust-sensing module was placed on the top side of the quadrotor platform to minimize high velocity air flow that is fundamental to similar quadcopters [43,44]. Figure 3 shows the system architecture for the modular dust sensor in detail.

The system is constructed around an Arduino MEGA 2560, powered by a 7.4 V lithium polymer battery, data telemetry is via XBee Pro S1 (2.4 GHz) radio transmitter while a GP-635T provides a timestamp for serial port data. Sensors include a SEN51035P temperature and humidity sensor and GP2Y10 SHARP dust sensor (Figure 3). All data was transmitted and logged on a ground station which displays received raw values and PM10 concentration readings in real time.

### 3.3. UAV Platforms

Both a fixed-wing and a multi-rotor UAV were selected to develop the sensing system. These aerial vehicles operate in fundamentally different ways with fixed-wing UAVs traversing a set volume of air in a given time while the hovering ability of rotary-winged UAVs allow collection of data at specific locations in space and time; however, they experience wind.

**Figure 4:**
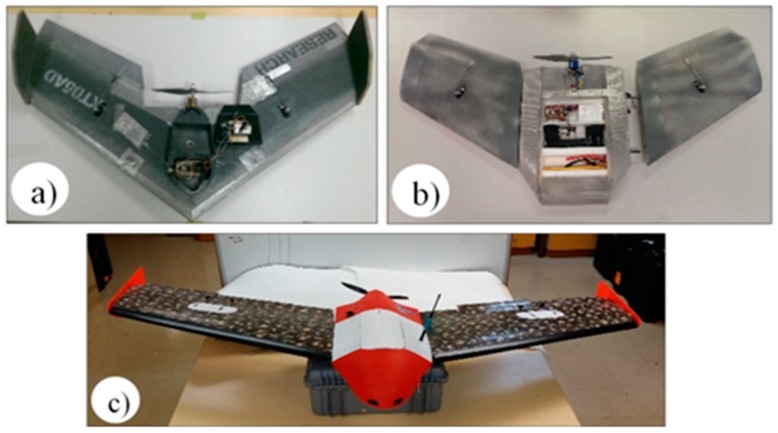
Fixed-wing UAV platforms, (**a**) Teklite; (**b**) GoSurv; and (**c**) Swamp Fox.

The suitability of three fixed-wing platforms was considered for integration with the optical and/or electrochemical sensors. All UAVs are constructed of expanded polypropylene (EPP) and composite materials that have been demonstrated as safe and robust platforms in the mining industry environment. Specifications for the models considered during this investigation are provided in Table 2 and Figure 4.

**Table 2:** Characteristics of UAVs identified as feasible platforms for this investigation.

Model	Wingspan (mm)	Length (mm)	Flying Weight (g)	Endurance (min)	Approx. Payload ^(3)^ (g)
Teklite ^(1)^	900	575	900–950	45	200
GoSurv ^(2)^	850	350	900–1200	50	>300
Swamp Fox [45] ^(1)^	1800	1000	4500	40	1000

^(1)^ Commercially available platform; ^(2)^ Fixed-wing platform designed at UQ SMI-CMLR; ^(3)^ Determined through experimental procedures.

All UAVs listed in Table 2 have low kinetic energy (<50 joules) and low air speed (<60 km/h). Low kinetic energy and speed improve safety and simplifies data acquisition performance but require reasonably calm conditions to operate. All have a pusher-propeller design providing access to clean airflow for sensors. The Paparazzi autopilot used on Teklite and GoSurv records altitude, platform coordinates, speed and direction. They also estimate wind speed and direction by response difference. The autopilot of the Swampfox platform records airspeed using a pitot tube, as well as speed and direction with the GPS. Air speed and geolocation data are drawn from the autopilot telemetry that is integral to all small UAV operations.

The Teklite was selected as the best platform for the type of test to be conducted due to its portability, ease of integration of sensors, successful flight testing, light weight and low (<100 ft) target flight altitude. The UAV is controlled using a ground control station. The flight plan is preloaded from the ground station that displays the flight parameters of the UAV, flight route and atmospheric pollution readings in real time. The flight plan can also be modified manually using a handheld radio transmitter and/or by altering the parameters through the ground station.

The UAV can be flown from as far as 1.5 km from the ground control station. Weather conditions (wind speed and direction, temperature, *etc.*) are used to pre-plan the UAV flight path to follow and characterize the blasting plume. If required, a flight path can be modified and uploaded into the ground station based on post-blasting observations. The UAV is restricted to fly more than 35 m above ground level as a safety factor to avoid collision with trees or infrastructure.

**Figure 5:**
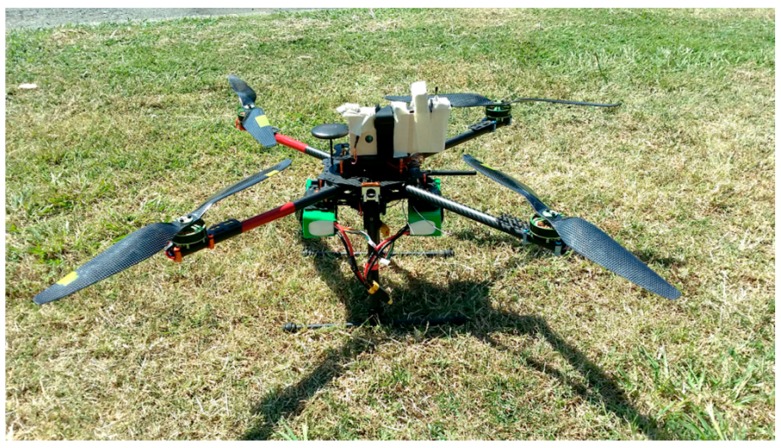
Quadcopter and modular gas-sensor system integrated.

A multi-rotor platform was used to record readings below 35 m above ground level. The system was designed for agricultural and air-monitoring surveys. Figure 5 shows the quadcopter integrated with the modular dust sensor. The multi-rotor platform has an average flight time of 20 min and a total weight of 2.5 kg (with batteries). The modular dust sensor had a total weight of 150 g and was placed inside a plywood case.

## 4. Bench Testing of Optical Sensors

**Figure 6:**
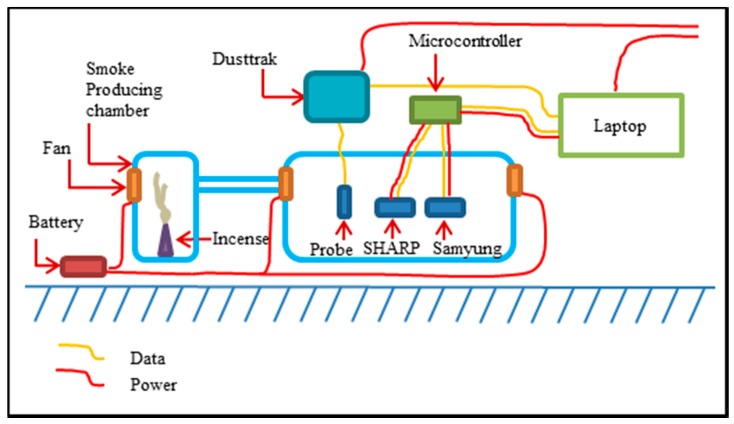
Gas chamber for sensor testing and calibration.

A gas chamber (see Figure 6) based on the work of Budde *et al.* [46] was constructed in order to expose the sensor node to different concentrations of particles and compare the readings against a calibrated dust-monitoring device—Dusttrak 8520. The Dusttrak has a response time of 1 s, a resolution of 0.001 mg/m^3^ and is capable of monitoring PM10 and PM2.5. Smoke from standard incense sticks was used as an airborne particulate source. The Samyoung sensor produced a low correlation coefficient (R^2^) of 0.5 and was therefore deemed an unsuitable option for the integration of the dust-monitoring module and UAVs.

### Results for SHARP (GP2Y10)

**Figure 7:**
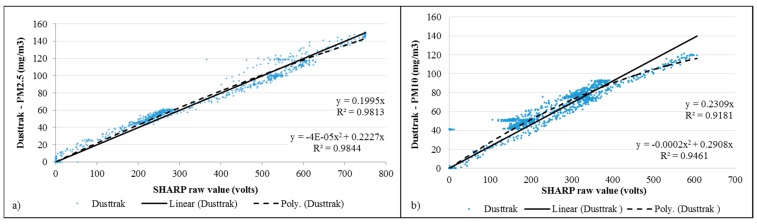
Correlation of raw values obtained with SHARP sensor for (**a**) PM2.5 and (**b**) PM10 *vs*. readings collected with Dusttrak (mg/m^3^).

Tests for PM10 and PM2.5 where undertaken for the SHARP dust sensor. An initial data collection test was used to correlate the raw values obtained from the sensor, which is the voltage modified by the light absorption of the receiver, with the values registered by the Dusttrak (see Figure 7). A linear and second-degree calibration equation, with correlation coefficients greater than 0.9, were obtained and applied to the sensor data.

**Figure 8:**
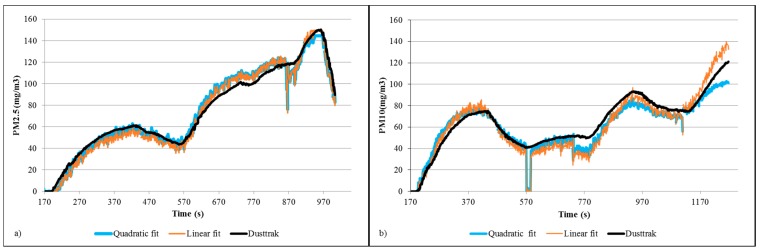
Linear and quadratic linear fit for raw SHARP values of (**a**) PM2.5 and (**b**) PM10 particle concentrations.

The original algorithm of M. Chardon and Trefois [47] developed to use the SHARP sensor with an Arduino board was modified to take readings every second. The objective of this test was to check that the data collected by the SHARP sensor was comparable to the Dusttrak readings, results are shown in Figure 8. The offset observed in the initial test was reduced having a satisfactory match between sensors. Percentage errors were calculated obtaining 38.0% and 13.6% for PM10 linear and quadratic fits respectively. PM2.5 errors were 11.9% and 9.96% for linear and quadratic fits respectively.

**Figure 9:**
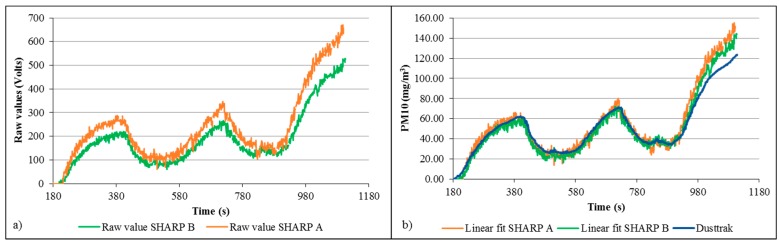
Dual SHARP and Dusttrak test showing (**a**) raw values data and (**b**) corrected particle measurements against Dusttrak readings.

A third test was done using two SHARP sensors and the Dusttrak (Figure 9). An offset between the SHARP sensors was also observed; however this error was reduced after correlating data with the linear equation fitted previously. For this test the linear fit produced a lower percentage error for SHARP A and B, of 19.3% and 12.5% respectively, however the second degree fit produced very similar results with errors of SHARP A:21.5% and SHARP B:14.9%.

Sensor variability due to temperature changes [5,30], was not considered for the experiment, however it will be undertaken in further tests.

## 5. Flight Test

### 5.1. SHARP Sensor Integrated to Fixed-Wing UAV

**Figure 10:**
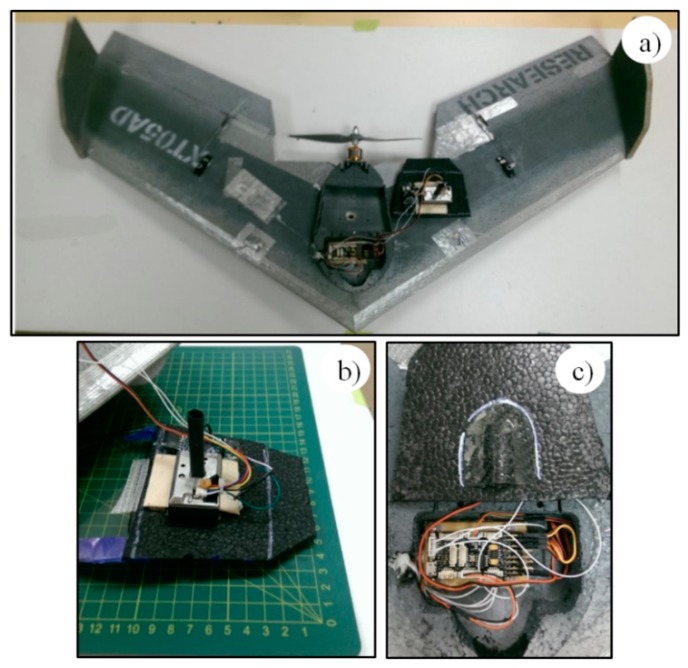
Modifications made to Teklite and SHARP sensor for flight, (**a**) Teklite UAV and SHARP sensor; (**b**) Air outlet for SHARP sensor; (**c**) Air intake for SHARP sensor.

The air intake and discharge were modified to produce a continuous flow inside the SHARP sensor chamber (see Figure 10). Air sampling intake was through a carbon fiber scooped cowl on the top surface of the wing directly over the sensor inlet. Sample exhaust was through a 4 mm tube attached to the sensor outlet and extended through the lower surface of the platform.

### 5.2. Test 1: Sensor Integration

**Figure 11:**
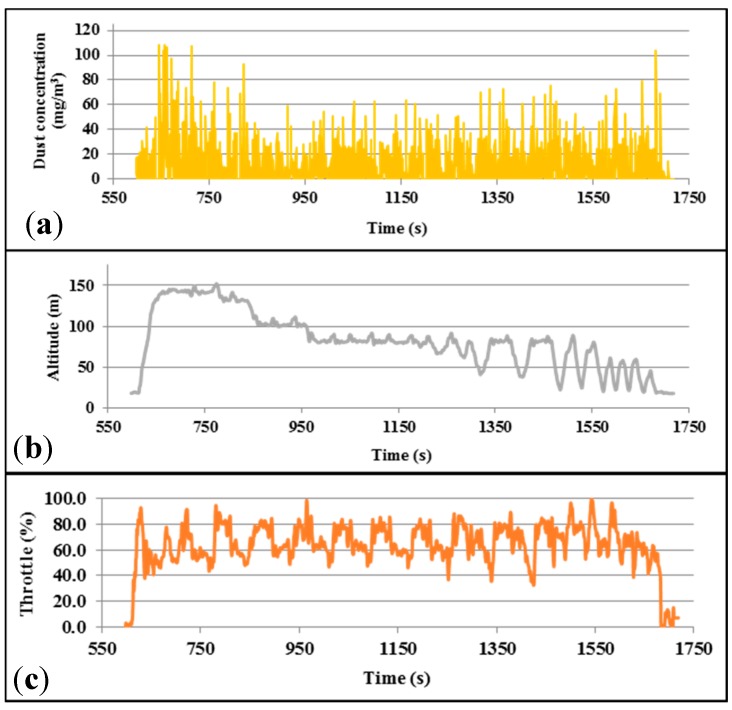
Data collected from Teklite flight with SHARP sensor attached. (**a**) Dust concentration; (**b**) Altitude; (**c**) Throttle.

**Figure 12:**
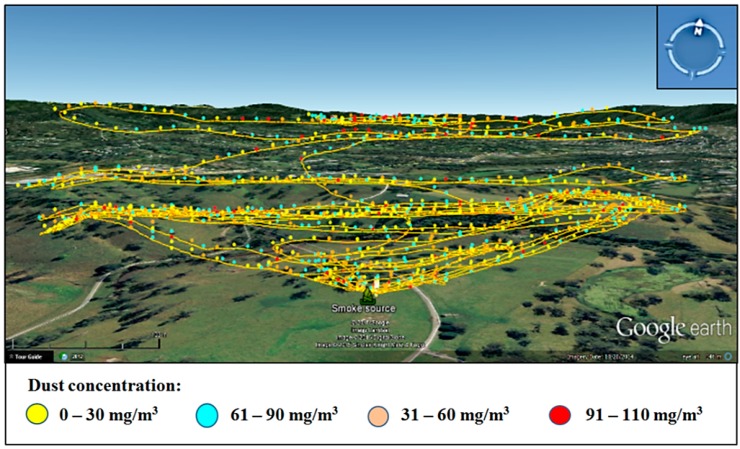
3D visualization of the Test 1 data collected with Teklite- SHARP sensor.

Several flights were made to test the feasibility of integrating the SHARP sensor with the Teklite platform. The first test was conducted on 6 June 2014 in order to evaluate the integration of the system. The test used a fire in an open area as an airborne particulate source. The UAV was programmed to fly around the fire for approximately 30 min. Data collected from the UAV and the air quality sensor are shown in Figure 11 and Figure 12. The data did not report variations in particulate matter concentration in the atmosphere, as it is observed in Figure 12. Analysis of the data indicated that electrical noise caused by motor and onboard electronics was interfering with the output.

High frequency noise consistent with electrical switching of motors and servos was filtered by installing a 50 V (0.1 µF) capacitor to the power source.

### 5.3. Test 2: PM10 Monitoring

A second field test was conducted on 13 October 2014 using “talcum powder” lifted into the atmosphere using a petrol-powered leaf blower (STIHL BG 56 Blower—max of 730 m^3^/h). Talcum powder was used due to its safe handling and availability. Talcum powder is composed of 0.2–0.3 mass fraction with a particle diameter no greater than 10 µm [48,49].

**Figure 13:**
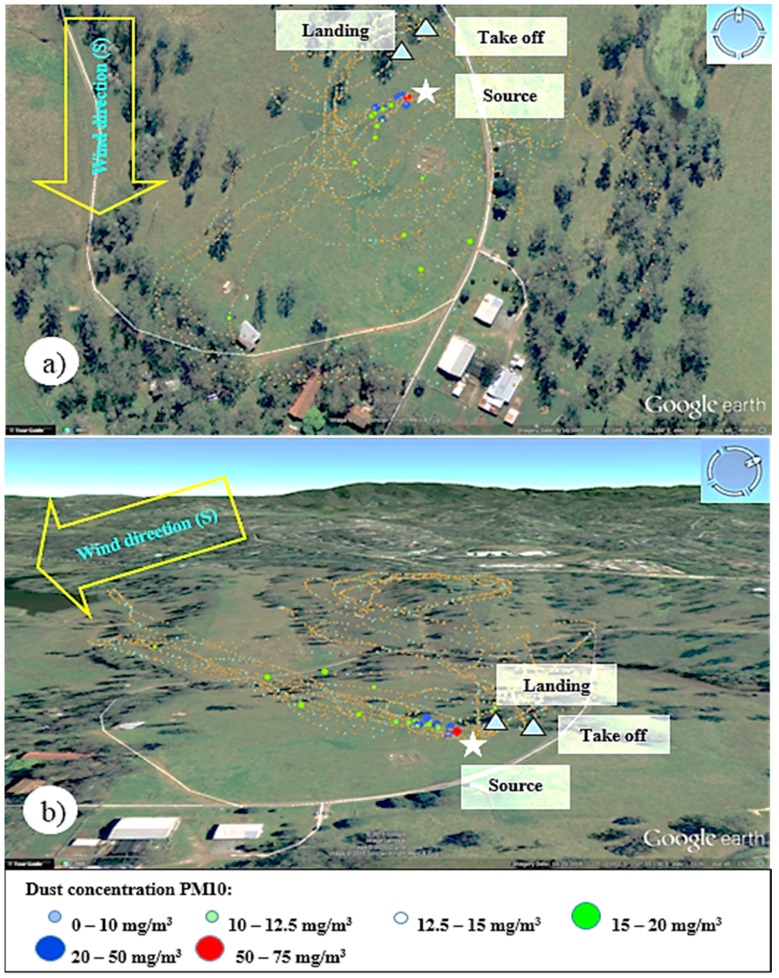
3D visualization of Test 2 data collected with Teklite-SHARP sensor for PM10 (**a**) Overview and (**b**) Side view.

In order to determine PM10 concentrations measured during the flight, the data was processed using the particle correlation (Figure 7) obtained from laboratory testing of the SHARP sensors. Figure 13 shows the distribution of PM10 concentrations in the atmosphere registered by the optical sensor by using top and side 3D visualization of the particulate plume. The wind direction was towards the west−southwest and concentrations ranged from 15 mg/m^3^ to 66 mg/m^3^, describing the shape of the plume when dispersed by the wind.

Tests 1 and 2 demonstrated the functionality and feasible integration of the system; however, the need for systematic characterization of a particulate plume of known composition and size remained. This is required to demonstrate the ability to calculate particulate emission rates, as most parameters can be independently measured using a constant powder emission, constant emission rate, known atmospheric conditions and particle size distribution of the source.

To achieve a systematic plume characterization, it was necessary that the UAV reproduced a fixed experimental flight pattern to aid spatial calculations and also exclude biased measurements that could easily be made when flying manually into the visible plume produced by the powder ejected. A flight path consisting of concentric circles at different heights and radius was planned for Test 3. The flight path ensures the UAV covers the designated area around the source. This ensures that the sensor intersects the plume and tests the ability of the data to describe the behavior of the plume in the air space surrounding it.

### 5.4. Test 3: Mixed Fixed and Rotary Wings

Test 3 was undertaken on 3 March 2015. The setup for the field experiment was based on Test 2, incorporating modifications to satisfy UAV flight and rigorous plume modelling requirements. The fixed-wing and multi-rotor UAVs were able to fly following the patterns programmed for the tests. Table 3 shows the radius and heights used for the test. These parameters were defined according to the capabilities of each UAV and to collect complementary datasets at two spatial scales.

**Table 3:** Programmed flight parameters and UAV capabilities.

Parameters	Quadcopter	Fixed-Wing
Max. Height *	120 m	120 m
Max. Radius	100 m	200 m
Programmed Heights (MAGL)	7, 14, 21	35, 45, 55
Programmed Radius	5, 15, 35	45, 55, 65, 75, 85

* Determined by UAV height flight restrictions [50].

**Figure 14:**
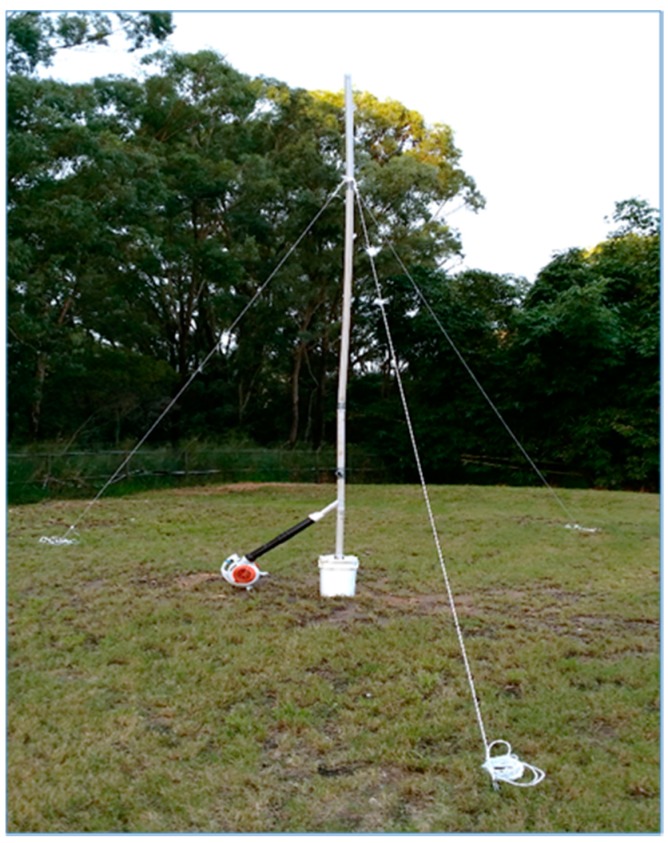
Powder ejection system setup.

The talcum powder plume was generated using a petrol-powered fan connected to a 5.5 m long and 0.05 m diameter PVC stack. The powder was loaded into the airstream through an intersection custom made for the powder containers at an approximate rate of 300 g/min (Figure 14).

**Figure 15:**
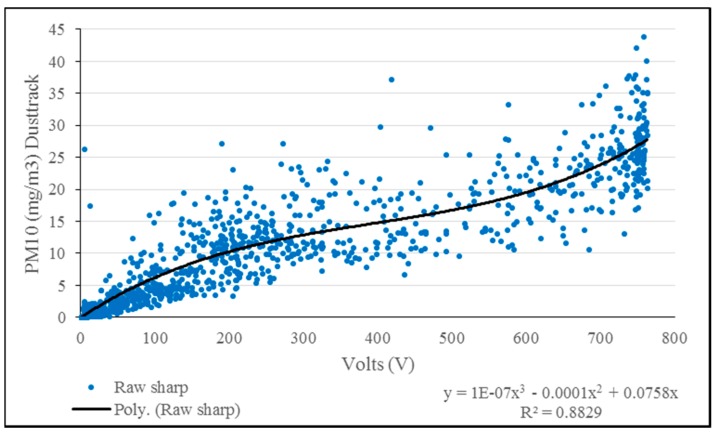
Correlation between talcum powder particles and raw value readings from the SHARP sensor and Dusttrak.

**Figure 16:**
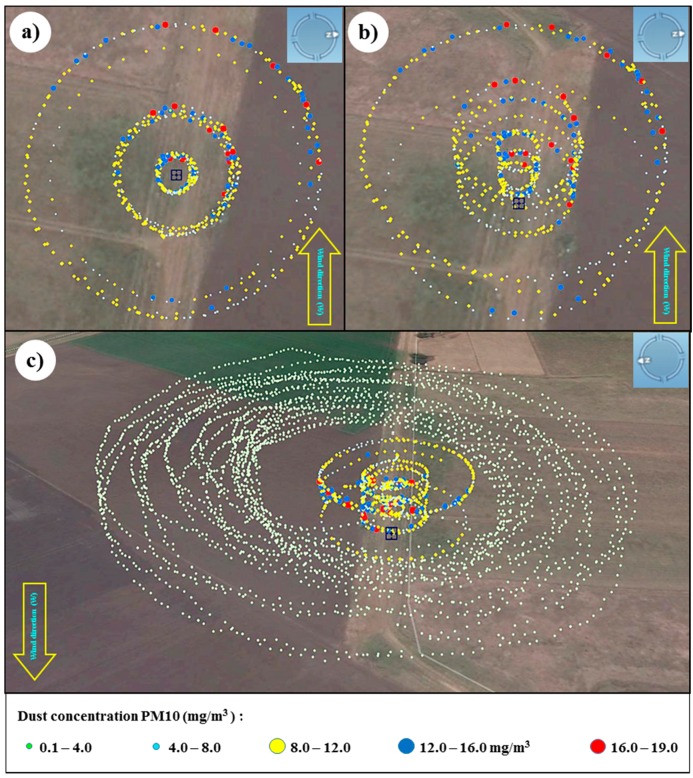
Flight path and PM10 concentrations monitored with the UAV quadcopter (**a**) top view and (**b**) side view; and (**c**) fixed-wing and quadcopter (overlapped flights).

For Test 3, the SHARP sensors where recalibrated due to the different characteristics including color and particle diameter that smoke and talcum powder have. The calibration procedure previously used for smoke particles was repeated for the talcum powder. A correlation equation was calculated using a polynomial fit by processing the data obtained with the Dusttrak 8520 and with the SHARP sensor (Figure 15). Integrated datasets from each platform were post-processed to visualize the concentrations measured by the fixed-wing and quadcopter during experimental flights (Figure 16).

PM10 concentration values ranged from 0.5 mg/m^3^ to 19 mg/m^3^ and their distribution described the path followed by the powder plume to the west, downwind from the source (Figure 16a,b). Mid-range concentrations to the east (downwind) and north of the source are likely the result of petrol motor exhaust particles and potentially spilled talcum powder. Experimental equipment modification using battery-powered fans and venture effect powder loading are being developed.

Future tests will also include measurements of background levels during flight monitoring periods to determine their influence in the UAV readings.

For safety reasons and the complexity involved in flying two UAVs simultaneously, the quadcopter and the fixed-wing UAVs were not flown simultaneously. The fixed-wing UAV was flown after the quadcopter and recorded maximum concentrations of 2.0 mg/m^3^ without an observable pattern (Figure 16c). Weather conditions with wind speed ranging from 7 to 9 m/s prevented the powder plume rising to the minimum programmed height of 35 m; therefore, it is unlikely that detectable particulates associated with the plume were present.

**Figure 17:**
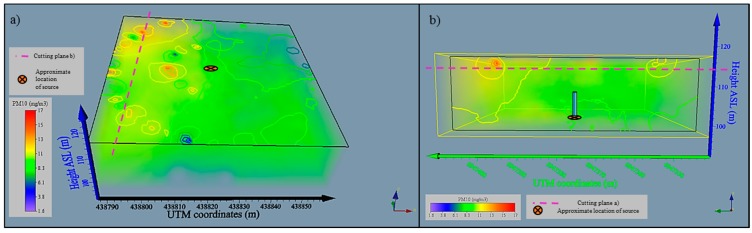
Volume rendering and contour plots created with quadcopter dataset (**a**) top view 18 m above ground level (from the East) and (**b**) side view 30 m away from the source (from the west).

Figure 17 shows the contour plots of the powder distribution at a height of 18 m above ground level and 30 m to the west of the source. The contour plots together with the volume rendering produced with the software Voxler aid in the interpretation of the data. They produce a model of the plume which can be challenging to interpret when plotting all readings independently, due to the high density of information. Higher concentrations of PM10 particles are shown in red color which are located in the western side of the source located at the center of the plot.

## 6. Conclusions and Further Work

The sensor systems developed to date are technically capable of delivering data comparable to industrial quality dust-monitoring devices but require individual calibration equations for each sensor used to characterize dust plumes. The use of talcum powder is primarily a detection exercise at this stage as most particulate matter in this product has a diameter greater than 10 μm. System testing at PM2.5 will require a chemical source such as a smoke generator.

The tests described in this paper only measured concentrations with a precision of 1 mg/m^3^; more precise readings of smaller concentrations will require the use of a different optical sensors and reference calibration with more precise equipment. Cross-contamination sources will require to be controlled in further experiments, and background levels will need to be measured to determine their content percentage in the final concentrations. These measurements will allow the programming of different flight patterns which could be focused in the intersection of the plume and will provide additional flight time.

Current experimental work indicates that integration of air quality sensor and autopilot data is feasible and will characterize airborne particulates in time and space.

Further work will be focused on the analysis of near-real time data to feed atmospheric modeling software and for flight path-planning algorithms.

## References

[1] Department of Natural Resources and Mines (DNRM) (2014). Queensland Monthly Coal Report.

[2] Raj R. (2005). Sustainable mining systems and technologies. Sustainable Mining Practices.

[3] NSWEPA EPA Investigating Reports of Blasting Fumes from Wambo Coa. http://www.epa.nsw.gov.au/epamedia/EPAMedia14051501.htm.

[4] Attalla M., Day S., Lange T., Lilley W., Morgan S. (2007). NOx Emissions from Blasting in Open Cut Coal Mining in the Hunter Valley.

[5] Sharp Opto-Electronic Devices Division Electronic Components Group. http://www.dema.net/pdf/sharp/PC847XJ0000F.pdf.

[6] SYhitech DSM501A Dust Sensor Module. http://i.publiclab.org/system/images/photos/000/003/726/original/tmp_DSM501A_Dust_Sensor630081629.pdf.

[7] Roy S., Adhikari G., Renaldy T., Singh T. (2011). Assessment of atmospheric and meteorological parameters for control of blasting dust at an Indian large surface coal mine. Res. J. Environ. Earth Sci..

[8] Roy S., Adhikari G.R., Singh T.N. (2010). Development of Emission Factors for Quantification of Blasting Dust at Surface Coal Mines. J. Environ. Protect..

[9] Roy S., Adhikari G., Renaldy T., Jha A. (2011). Development of multiple regression and neural network models for assessment of blasting dust at a large surface coal mine. J. Environ. Sci. Technol..

[10] Bridgman H., Carras J.N. (2005). Contribution of Mining Emissions to NO_2_ and PM10 in the Upper Hunter Region.

[11] Richardson C. (2013). PM2.5 Particulate Emission Rates From Mining Operations.

[12] Koronowski R. FAA Approves Use of Drones by ConocoPhillips to Monitor Oil Drilling Activities in Alaska. http://thinkprogress.org/climate/2013/08/26/2524731/drones-conocophillips-alaska/.

[13] Fernandez R. Methane Emissions from the U.S. Natural Gas Industry and Leak Detection and Measurement Equipment.

[14] Nicolich K. High Performance VCSEL-Based Sensors for Use with UAVs. http://www.princeton.edu/pccmeducation/undergrad/reu/2012/Nicolich.pdf.

[15] DECCW Upper Hunter Air Quality Monitoring Network. www.environment.nsw.gov.au/aqms/upperhunter.htm.

[16] DEHP Air Quality. http://www.ehp.qld.gov.au/air/.

[17] (2014). Dräger X-am^®^ 5600. http://www.draeger.com/sites/assets/PublishingImages/Products/cin_x-am_5600/UK/9046715_PI_X-am_5600_EN_110314_fin.pdf.

[18] Picarro (2014). PICARRO Surveyor.

[19] Picarro, Picarro (2015). Picarro G2401 CO_2_ + CO + CH_4_ + H_2_O CRDS Analyzer.

[20] QLDGov Tapered Element Oscillating Microbalance. https://www.qld.gov.au/environment/pollution/monitoring/air-pollution/oscillating-microbalance/.

[21] ThermoScientific Thermo Scientific TEOM^®^ 1405-DF. http://www.thermo.com.cn/Resources/200802/productPDF_3275.pdf.

[22] Geotech AQMesh Operating Manual. http://www.geotechuk.com/media/215152/aqmesh_operating_manual.pdf.

[23] LAvionJaune Ultra-Light, Standalone Lidar System for UAVs (Laser Scanner, IMU, RTKGPS, Processing Unit). http://yellowscan.lavionjaune.com/data/leafletYS.pdf.

[24] Sihler H., Kern C., Pöhler D., Platt U. (2009). Applying light-emitting diodes with narrowband emission features in differential spectroscopy. Opt. Lett..

[25] Kern C., Trick S., Rippel B., Platt U. (2006). Applicability of light-emitting diodes as light sources for active differential optical absorption spectroscopy measurements. Appl. Opt..

[26] Choi S., Kim N., Cha H., Ha R. (2009). Micro Sensor Node for Air Pollutant Monitoring: Hardware and Software Issues. Sensors.

[27] Thalman R.M., Volkamer R.M. (2009). Light Emitting Diode Cavity Enhanced Differential Optical Absorption Spectroscopy (led-ce-doas): A Novel Technique for Monitoring Atmospheric Trace Gases. Proc. SPIE.

[28] Khan A., Schaefer D., Roscoe B., Kang S., Lei T., Miller D., Lary D.J., Zondlo M.A. Open-path greenhouse gas sensor for UAV applications. Proceedings of the 2012 Conference on Lasers and Electro-Optics (CLEO).

[29] Malaver A., Gonzalez F., Motta N., Depari A., Corke P. Towards the Development of a Gas Sensor System for Monitoring Pollutant Gases in the Low Troposphere Using Small Unmanned Aerial Vehicles. Proceedings of Workshop on Robotics for Environmental Monitoring, Sydney University.

[30] Neumann P.P., Hernandez Bennetts V., Lilienthal A.J., Bartholmai M., Schiller J.H. (2013). Gas source localization with a micro-drone using bio-inspired and particle filter-based algorithms. Adv. Robot..

[31] Bennetts V.H., Lilienthal A.J., Neumann P.P., Trincavelli M. (2011). Mobile robots for localizing gas emission sources on landfill sites: Is bio-inspiration the way to go?. Front. Neuroeng..

[32] Watai T., Machida T., Ishizaki N., Inoue G. (2005). A Lightweight Observation System for Atmospheric Carbon Dioxide Concentration Using a Small Unmanned Aerial Vehicle. J. Atmos. Ocean. Technol..

[33] Brown J., Taras M. (2008). Remote Gas Sensing of SO_2_ on a 2D CCD (Gas. Camera).

[34] Lega M., Napoli R.M.A., Persechino G., Kosmatka J. New techniques in real-time 3D air quality monitoring: CO, NO_x_, O_3_, CO_2_, and PM. Proceedings of the NAQC 2011.

[35] Lega M., Kosmatka J., Ferrara C., Russo F., Napoli R.M.A., Persechino G. (2012). Using Advanced Aerial Platforms and Infrared Thermography to Track Environmental Contamination. Environ. Forensics.

[36] Saghafi A., Day S., Fry R., Quintanar A., Roberts D., Williams D., Carras J.N. Development of an Improved Methodology for Estimation of Fugitive Seam Gas. Emissions from Open Cut Mining. http://www.acarp.com.au/abstracts.aspx?repId=C12072.

[37] Gonzalez F., Castro M.P., Narayan P., Walker R., Zeller L. (2011). Development of an autonomous unmanned aerial system to collect time-stamped samples from the atmosphere and localize potential pathogen sources. J. Field Robot..

[38] Gonzalez L.F., Castro M.P., Tamagnone F.F. Multidisciplinary design and flight testing of a remote gas/particle airborne sensor system. Proceedings of the 28th International Congress of the Aeronautical Sciences, Optimage Ltd., Brisbane Convention & Exhibition Centre.

[39] Malaver A., Motta N., Corke P., Gonzalez F. (2015). Development and Integration of a Solar Powered Unmanned Aerial Vehicle and a Wireless Sensor Network to Monitor Greenhouse Gases. Sensors.

[40] Reed W.R. (2005). Significant Dust Dispersion Models for Mining Operations.

[41] Stockie J.M. (2011). The Mathematics of Atmospheric Dispersion Modeling. SIAM Rev..

[42] Visscher A.D. (2013). An Air Dispersion Modeling Primer. Air Dispersion Modeling.

[43] Roldán J.J., Joossen G., Sanz D., del Cerro J., Barrientos A. (2015). Mini-UAV Based Sensory System for Measuring Environmental Variables in Greenhouses. Sensors.

[44] Haas P., Balistreri C., Pontelandolfo P., Triscone G., Pekoz H., Pignatiello A. Development of an unmanned aerial vehicle UAV for air quality measurements in urban areas. Proceedings of the 32nd AIAA Applied Aerodynamics Conference; American Institute of Aeronautics and Astronautics.

[45] Skycam Swamp Fox UAV. http://www.kahunet.co.nz/swampfox-uav.html.

[46] Budde M., ElMasri R., Riedel T., Beigl M. (2013). Enabling Low-Cost Particulate Matter Measurement for Participatory Sensing Scenarios. Proceedings of the 12th International Confrence on Moile and Ubiquitous Multimedia MUM.

[47] M.Chardon C., Trefois C. (2012). Standalone Sketch to Use with a Arduino Fio and a Sharp Optical Dust Sensor GP2Y1010AU0F.

[48] Fiume M.M. (2013). Safety Assessment of Talc As Used in Cosmetics.

[49] Klingler G.A. (1972). Digital Computer Analysis of Particle Size Distribution in Dusts and Powders.

[50] CASA (1998). Civil Aviation Safety Regulations 1998. Unmanned Air and Rockets.

