# Correction: Alvarado, M., et al. Towards the Development of a Low Cost Airborne Sensing System to Monitor Dust Particles after Blasting at Open-Pit Mine Sites. *Sensors* 2015, *15*, 19667–19687

**DOI:** 10.3390/s16071028

**Published:** 2016-07-05

**Authors:** Miguel Alvarado, Felipe Gonzalez, Andrew Fletcher, Ashray Doshi

**Affiliations:** 1 Centre for Mined Land Rehabilitation, Sustainable Mineral Institute, The University of Queensland, Brisbane 4072, Australia; a.fletcher@cmlr.uq.edu.au; 2 Science and Engineering Faculty, Queensland University of Technology (QUT), Brisbane 4000, Australia; felipe.gonzalez@qut.edu.au; 3 Faculty of Engineering, Architecture and Information Technology, School of Information Technology and Electrical Engineering, The University of Queensland, St. Lucia 4072, Australia; ashraydoshi@gmail.com

The author wishes to change Figure 1 and Figure 3 from his paper published in *Sensors* [1], doi:10.3390/s150819667, website: http://www.mdpi.com/1424-8220/15/8/19667 for Figure 1 and Figure 2 presented in this ‘Correction’.

**Figure 1:**
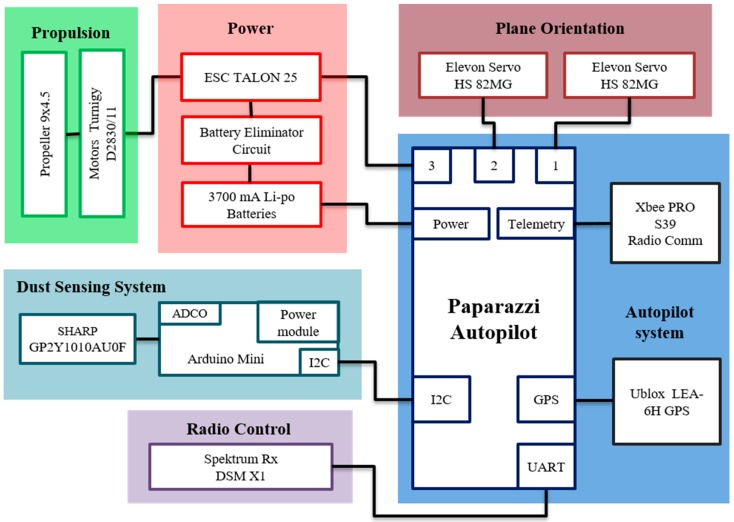
System architecture for the fixed-wing UAV with dust sensor.

**Figure 2:**
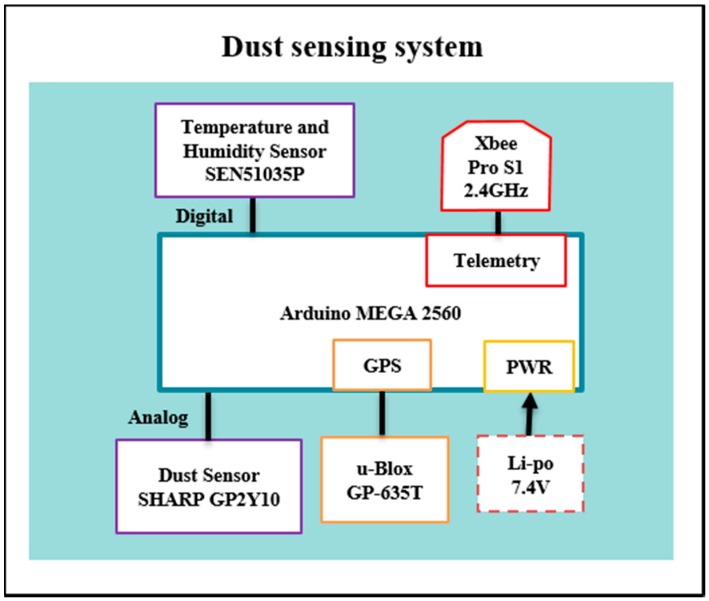
System architecture for the modular dust sensor.
